# Non-Proliferative Immune Restoration by *Euglena gracilis* EK Powder in Cyclophosphamide-Induced Immunosuppressed Mice

**DOI:** 10.4014/jmb.2607.07031

**Published:** 2026-09-09

**Authors:** Seung Woo Ahn, Yun Hwan Park, Chang Ho Cho, Seung Woo Jo, Mi Gyeong Lee, Yoon-E Choi

**Affiliations:** 1 Division of Environmental Science and Ecological Engineering, Korea University, Seoul 02841, Republic of Korea; 2 OJeong Resilience Institute, Korea University, Seoul 02841, Republic of Korea; 3 Institute of Life Science and Natural Resources, Korea University, Seoul 02841, Republic of Korea; 4 Euealbiotech Co., Ltd., Seoul 03980, Republic of Korea

**Keywords:** *Euglena gracilis*, Paramylon, Cyclophosphamide, Immunomodulation, Natural killer cell, Immunoglobulin

## Abstract

Cytotoxic chemotherapy frequently induces immunosuppression, impairing the innate cellular and humoral immune responses. Therefore, immunomodulatory agents that restore immune function without indiscriminate leukocyte expansion are required. This study examined whether *Euglena gracilis* EK powder (EKP), a paramylon-rich β-1,3-glucan preparation, supports immune recovery under cyclophosphamide (CPA)-induced immunosuppression. Male BALB/c mice received EKP (80, 200, or 500 mg/kg/day for 21 days) under a dual-temporal CPA design: acute phase (days 7 and 9) and established phase (days 14 and 17). The immune status was assessed using peripheral hematology, splenocyte viability, cytokine profiling, natural killer (NK) cell-associated activity, serum immunoglobulin levels, and splenic histopathology. EKP did not restore the peripheral leukocyte counts in either model, indicating that its immunological effects were independent of hematopoietic recovery. However, EKP treatment improved the levels of functional immune markers in a dose-dependent manner. EKP increased basal splenocyte viability without enhancing mitogen-induced proliferation, restored IFN-γ and TNF-α production, and suppressed excessive IL-6 levels. NK cell-associated activity recovered across all EKP doses (*p* < 0.05–0.01), consistent with the increase in IL-12 p70 levels at the highest dose. Serum IgG levels were restored at all doses (*p* < 0.05–0.01); however, IgM and IgA showed class-specific recovery at 200 and 500 mg/kg, respectively, with IgA recovery suggesting possible gut-associated lymphoid tissue involvement. Splenic histopathology confirmed the dose-dependent recovery of white pulp organization and germinal center formation. These findings support a framework wherein EKP improves immune functional markers by preserving existing immune components and cytokine rebalancing, rather than increasing circulating leukocytes.

## Introduction

The immune system orchestrates innate, cellular, and humoral responses to defend against pathogens and maintain physiological homeostasis, and its impairment increases the susceptibility to infection and chronic diseases. In modern society, the burden of immune dysfunction has grown because of aging, stress, and lifestyle-related factors, heightening the need for safe strategies that restore immune competence rather than merely amplifying immune cell numbers. Immunosuppression can arise from various causes, of which chemotherapy is a clinically significant example. Chemotherapy-induced immunotoxicity poses a major clinical challenge, as it compromises not only the number of circulating immune cells but also the coordinated function of innate, cellular, and humoral immune responses. Cyclophosphamide (CPA), a widely used alkylating agent, induces immunosuppression through its DNA alkylating metabolites, to which rapidly proliferating lymphocytes are particularly susceptible [1]. As a result, CPA exposure leads to lymphopenia, impaired natural killer (NK) cell activity, cytokine dysregulation, reduced immunoglobulin production, and disruption of lymphoid organ architecture [1, 2]. These features render CPA-induced immunosuppression a well-validated experimental model for evaluating agents aimed at restoring immune competence in chemotherapy-associated conditions.

Restoring immune function in this context presents its own pharmacological challenge because simply stimulating immune cell proliferation is not always a safe or effective strategy. Conventional immunostimulatory approaches can drive leukocyte expansion; however, this may be accompanied by hematological imbalance or exacerbated inflammation [2]. A more targeted approach would involve agents that improve the functional capacity of existing immune cell populations, rather than simply increasing cell numbers. This distinction is especially important in chemotherapy-induced immunosuppression, in which functional immune deficits frequently persist even in the absence of measurable leukocyte depletion.

*Euglena gracilis*, a photosynthetic freshwater microalga, produces paramylon as its primary intracellular carbohydrate reserve [3, 4]. Paramylon is a high-molecular-weight, linear β-1,3-glucan with an unbranched homopolymeric structure [5], a feature that distinguishes it from the branched β-glucans found in yeast or fungi and that may influence how it interacts with immune cells. Specifically, paramylon can engage pattern recognition receptors, including Dectin-1 and TLR2/6, expressed on myeloid cells [6, 7], thereby providing a mechanistic basis for modulating innate immune signaling pathways such as NF-κB and MAPK activation, cytokine production, and downstream NK cell responses [8]. Building on this immunopharmacological basis, *E. gracilis* EK powder (EKP) has emerged as a candidate natural immunomodulator with the potential to regulate immune homeostasis. In the present study, whole EKP, rather than purified paramylon, was evaluated, reflecting the aim of characterizing a commercially produced, application-ready material in which paramylon is the major, but not sole, constituent.

Previous studies have reported that EKP and paramylon can enhance NK cell activity and modulate cytokine-related pathways in both CPA-immunosuppressed mice and healthy human subjects [9, 10]. However, these investigations have largely focused on individual immune endpoints, leaving a broader immunological picture uncharacterized. In particular, the integrated relationship between hematological recovery, splenocyte viability, cytokine network regulation, NK cell function, humoral immunity, and lymphoid tissue architecture has not yet been systematically examined. It remains unclear whether the EKP-mediated immune recovery reflects hematopoietic expansion or functional reprogramming of the existing immune cell pool, a distinction that is central to defining the pharmacological mode of action of EKP in chemotherapy-induced immunosuppression.

To address this gap, the present study evaluated the dose-dependent immunorestorative effects of EKP in CPA-induced immunosuppressed mice using a dual-temporal model designed to capture both the acute and established phases of immune disruption. Based on comprehensive immune profiling, we aimed to determine whether EKP restores immune homeostasis through the functional modulation of existing immune cells rather than hematopoietic expansion. We hypothesized that EKP would support splenocyte survival, restore disrupted cytokine responses and NK cell-associated responsiveness, improve humoral immune markers, and promote splenic architectural recovery, a pattern collectively consistent with a non-proliferative immunorestoration framework.

## Materials and Methods

### Experimental Design and *In Vivo* Temporal Models

Seven-week-old male SPF BALB/c mice (Samtako Bio Korea Co., Ltd., Republic of Korea) were housed under controlled conditions (22 ± 3°C, 50 ± 20% RH, 12-h light/dark cycle) with access to irradiated chow and water *ad libitum*, in accordance with established guidelines [11]. All animal experiments were conducted at the Korea Conformity Laboratories, an independent accredited testing facility, and were approved by the Institutional Animal Care and Use Committee (IACUC approval no. NT24-00194). At 8 weeks, mice were weight-stratified into six groups (n = 10 per group): G1 (vehicle); G2 (CPA control); G3–G5 (CPA + EKP at 80, 200, and 500 mg/kg/day); and G6 (CPA + β-glucan at 400 mg/kg/day). EKP (lot no. 405153; Euealbiotech Co., Ltd., Republic of Korea) was produced from heterotrophically cultivated *E. gracilis* biomass under conditions optimized for commercial-scale manufacturing rather than for maximizing paramylon accumulation. Quantitative analysis showed that paramylon, a linear β-1,3-glucan, was the major component, accounting for approximately 30% (w/w) of the dry powder, with the remainder consisting of other *Euglena*-derived cellular constituents such as proteins, lipids, and minerals. This composition was consistently maintained across the production batches during the established manufacturing process. EKP and β-glucan (Naturence Co., Ltd., Republic of Korea) were prepared in sterile distilled water and administered via daily oral gavage (10 mL/kg/day) for 21 days. β-Glucan was used as a positive control, as it is an established immunomodulatory reference material approved by the Korean Ministry of Food and Drug Safety as a functional ingredient for immune enhancement (approval no. 2020-10), and the 400 mg/kg/day dose follows previous studies employing β-glucan at this dose as a positive control in comparable *Euglena*/CPA mouse models [10, 12]. Immunosuppression was induced using two intraperitoneal injections of cyclophosphamide (150 mg/kg; Merck KGaA, Germany; Cat. No. C0768). Two temporal models were employed to capture distinct immunosuppressive dynamics: Trial 1 (acute phase) with CPA administered on days 7 and 9, and Trial 2 (established phase) with CPA delayed to days 14 and 17. Clinical signs and body weights were monitored weekly throughout the 21-day period [13].

### Systemic Hematological and Humoral Profiling

On Day 21, the mice were anesthetized with isoflurane and blood was drawn from the abdominal aorta. For hematological analysis, whole blood collected in EDTA-K2 microtainers (#365974; Becton, Dickinson and Company, USA) was processed using an automated analyzer (ADVIA 2120; Siemens Healthineers) to determine total white blood cell (WBC) and lymphocyte (LY) counts (Trial 1), along with a complete differential count of neutrophils, eosinophils, basophils, monocytes, and reticulocytes (Trial 2). For humoral profiling (Trial 2), whole blood was allowed to clot for 30 min and centrifuged (1,500 × g, 10 min, 4°C). The resultant serum was stored at -80°C until analysis. Serum concentrations of IgA, IgM, and IgG were subsequently quantified using commercial ELISA kits (IgA: #AB157717; IgM: #AB133047, 1:15,000 dilution; IgG: #AB151276, 1:5,000 dilution; Abcam, UK), with absorbance measured at 450 nm.

### Primary Splenocyte Isolation and Multi-modal Functional Assays

Following exsanguination, spleens were aseptically excised and weighed. To preserve functional integrity, minced splenic tissue was enzymatically dissociated in HBSS containing Collagenase IV (100 U/mL; #17104019; Gibco, USA) and DNase I (20 U/mL; #90083; Thermo Fisher Scientific, USA) at 37°C for 20–30 min [14]. Following 1 mM ethylenediaminetetraacetic acid (EDTA) arrest and RBC lysis (#00-4300-54; eBioscience, USA), splenocytes were resuspended in complete RPMI 1640 (#11875093; Gibco, Thermo Fisher Scientific, USA). For functional assays, cells (2 × 10^6^/well) were cultured for 48 h to assess proliferation (CCK-8 assay) under unstimulated and ConA (1 μg/mL)-stimulated conditions [15]. Concurrently, cells were stimulated with anti-CD3/CD28 mAbs (1 μg/well) to quantify secreted cytokines via Quantikine ELISA (IL-6 [#M6000B-1], IFN-γ [#MIF00-1], TNF-α [#MTA00B-1], and IL-12 p70 [#M1270-1]; R&D Systems, USA). In Trial 1, IL-1β (#MLB00C-1), IL-4 (#M4000B-1), IL-10 (#M1000B-1), and IL-12 p70 levels were additionally assayed but were below the limit of quantification (< LOQ) in all groups; IL-12 p70 levels were therefore quantified only in Trial 2. IL-2 was also measured in Trial 1 but was not reported as no significant differences were observed between the CPA control and EKP-treated groups. For NK cell activity (Trial 1), cells (1 × 10^6^/well) were treated with an NK activator (#ATGBF001; NKMAX, Republic of Korea) for 24 h, using IFN-γ secretion as a surrogate cytotoxic index [16].

### Splenic Histopathology

Representative spleens (two per group) were fixed in 10% neutral buffered formalin, paraffin-embedded, and sliced into 4-μm thick sections. H&E-stained sections were examined under a microscope (Axiovert 5; Carl Zeiss AG, Germany) and evaluated by a board-certified veterinary pathologist to assess white pulp integrity, germinal center formation [17], and lymphocyte density.

### Statistical Analysis

Data are expressed as mean ± SD. Body weight data were analyzed using two-way repeated-measures ANOVA with Geisser-Greenhouse correction, followed by Dunnett's multiple comparison test against the normal control group (GraphPad Prism version 8.0.2; GraphPad Software, USA). For all other endpoints, differences between the normal control (G1) and CPA control (G2), and between the CPA control (G2) and positive control (G6) were evaluated using an independent samples *t*-test, with Levene's test applied prior to each comparison to determine the appropriate variance assumption. Differences between the CPA control (G2) and EKP treatment groups (G3–G5) were assessed using parametric multiple comparison procedures when the data satisfied assumptions of normality and homogeneity of variance, or non-parametric multiple comparison procedures when these assumptions were not met, in accordance with the standard operating procedures of the testing facility. All analyses were performed using SPSS software (version 12.0; IBM Corp., USA). Statistical significance was defined as *p* < 0.05 and *p* < 0.01.

## Results and Discussion

### Validation of CPA-Induced Immunosuppression Across Temporal Models

To establish an interpretable model of chemotherapy-induced immunosuppression, two CPA administration schedules were used to represent the different stages of immune disruption. As illustrated in Fig. 1A, CPA was administered during the early phase on days 7 and 9 in Trial 1 and during the late phase on days 14 and 17 in Trial 2. This design allowed for distinguishing between acute systemic responses and more established immunosuppressive conditions.

In Trial 1, the CPA control group (G2) showed lower body weight than the normal control group (G1) at weeks 2 and 3 (*p* < 0.01; Fig. 1B). This result indicated that early CPA treatment induced acute systemic toxicity, consistent with the cytotoxic effect of CPA on rapidly proliferating cells [2]. The EKP-treated groups (G3–G5) and the positive control group (G6) had already shown significantly lower body weights than G1 at Week 1, prior to CPA administration (*p* < 0.05–0.01; Fig. 1B), indicating baseline differences in weight distribution at group allocation; consequently, between-group body weight comparisons in Trial 1 should be interpreted with this initial imbalance taken into account. Although all groups (including G2) exhibited a decline in weight at week 2 followed by weight regain at week 3, this temporal recovery reflected the natural resolution of CPA-induced acute toxicity rather than an EKP-specific effect, as between-group differences persisted throughout the study. Overall, EKP did not reverse CPA-induced systemic weight loss under acute treatment conditions.

In Trial 2, body weight did not differ significantly between the normal control (G1) and CPA control group (G2) at any time point (all *p* > 0.05; Supplementary Fig. S1). The only significant inter-group difference was a modest reduction in the low-dose EKP group relative to G1 at Week 3 (*p* < 0.01), which did not follow a dose-related pattern and was not observed at higher doses. The absence of a CPA-induced weight loss indicated that late CPA administration induced immune suppression without producing overt systemic wasting under the present conditions. Trial 2 provided a model in which immune-related outcomes could be interpreted with less interference from generalized body weight loss [2]. The different body weight patterns in Trials 1 and 2 confirmed that the timing of CPA exposure strongly influenced the apparent systemic phenotype of the model.

The spleen weights were further distinguished between the two temporal models (Fig. 1C). In Trial 1, spleen weight was significantly higher in G2 than in G1 (*p* < 0.01). This increase may reflect an acute compensatory or inflammatory response following early CPA-induced immune injury [18]. In contrast, in Trial 2, spleen weight was markedly reduced in G2 compared to that in G1 (*p* < 0.01), indicating splenic atrophy associated with sustained immunosuppression [1]. These opposing spleen weight responses suggest that Trial 1 represents an acute, compensatory phase, whereas Trial 2 represents a more established immunosuppressed state.

Across both temporal conditions, EKP administration did not significantly alter spleen weight compared to that in the CPA control group. This finding indicated that EKP did not induce measurable lymphoid organ enlargement or gross organ-level recovery. Together with the body weight results, these data show that EKP does not act through general physiological restoration or lymphoid organ enlargement.

Overall, the dual-temporal CPA model successfully captured two distinct immunosuppressive states: an acute systemic response in Trial 1 and an established splenic atrophy-dominant state in Trial 2. The absence of body or spleen weight recovery following EKP treatment provides an important basis for interpreting the subsequent immune functional outcomes as effects that are not simply explained by whole-body recovery or lymphoid organ expansion.

### Limited Numerical Restoration of Peripheral Immune Cells Following EKP Treatment

To determine whether the EKP-mediated immune effects were associated with the numerical recovery of circulating immune cells, peripheral hematological profiles were analyzed in both temporal CPA models (Supplementary Table S1). In Trial 1, total WBC counts in the CPA control group (G2) showed a modest decrease compared to those in the normal control group (G1), whereas lymphocyte counts were significantly reduced (*p* < 0.01) [19]. This indicates that early CPA exposure primarily causes selective lymphoid depletion rather than generalized leukocyte reduction. The EKP-treated groups (G3–G5) and the positive control group (G6) did not recover WBC or lymphocyte counts compared to G2; rather, these values were further reduced (*p* < 0.05–0.01), indicating that EKP did not restore peripheral immune cell numbers during the acute phase of CPA-induced immunosuppression.

A similar pattern was observed in Trial 2, which represented a more established immunosuppressed state. CPA treatment significantly reduced WBC, neutrophil, eosinophil, lymphocyte, and reticulocyte counts compared to G1 (all *p* < 0.01) [20]. These changes confirm the induction of multilineage hematopoietic suppression by late CPA administration. Despite severe depletion, EKP administration did not restore any of the hematological parameters to normal levels. The low-dose EKP group (G3) showed a modest but significant increase in lymphocyte count relative to G2 (*p* < 0.05), although this effect was isolated and not sustained at higher doses. The direction of these minor differences was inconsistent between the two temporal models, further arguing against a systematic hematopoietic effect of EKP. These hematological results are important because they separate EKP-mediated immune recovery from leukocyte expansion. In both temporal models, EKP did not increase the number of circulating immune cells, suggesting that it does not act as a hematopoietic stimulant under the tested conditions. Therefore, any functional improvement observed in subsequent assays should not be attributed to increased peripheral leukocyte availability.

Instead, persistent leukocyte depletion provides a critical baseline for interpreting subsequent immune function data. As shown in the following sections, EKP improved basal splenocyte viability, cytokine responses, NK cell-associated activity, and serum immunoglobulin levels despite the absence of measurable peripheral leukocyte recovery. This dissociation between immune cell number and immune function supports the central interpretation that EKP promotes functional immune restoration without detectable expansion of circulating leukocytes.

Taken together, EKP did not restore peripheral leukocyte numbers in either temporal model. These findings indicate that EKP-associated immune recovery is unlikely to be driven by hematopoietic restoration but may involve functional preservation and reactivation of the remaining immune cell population.

### EKP Preserved Basal Splenocyte Viability without Inducing Proliferation

To determine whether EKP promotes immune cell survival under CPA-induced stress, basal splenocyte viability was assessed under unstimulated conditions (Fig. 2A). CPA treatment resulted in a numerical decrease in splenocyte viability in the CPA control group (G2) compared with that in the normal control group (G1), suggesting reduced splenocyte survival or metabolic activity following chemotherapy-induced immune injury. In contrast, EKP administration increased basal splenocyte viability in a dose-dependent manner. The low- and mid-dose groups showed progressive improvement, whereas the high-dose group (G5) and the positive control group (G6) exhibited significantly higher viability than G2 (*p* < 0.01), reaching or exceeding the levels observed in G1. This effect was observed under unstimulated conditions, indicating that EKP improved basal splenocyte status without requiring external mitogenic activation. This interpretation is consistent with the hematological results described above, in which EKP did not increase the number of circulating leukocytes.

To further examine whether the increase in basal viability reflected an enhanced proliferative capacity, splenocytes were stimulated with ConA (Supplementary Fig. S2). CPA treatment significantly reduced ConA-induced proliferation compared to G1. However, the EKP-treated groups did not show a significant recovery in mitogen-induced proliferation relative to G2. Similarly, the positive control group (G6) showed no enhancement but exhibited a significant reduction relative to G2 (*p* < 0.01). Thus, the improvement in basal splenocyte viability was not accompanied by restoration of T cell mitogenic proliferation.

The divergence between basal viability and stimulated proliferation provides important clues regarding the mode of action of EKP. EKP appeared to preserve or improve the viability of splenocytes remaining after CPA exposure, but did not drive proliferative expansion. Therefore, EKP-mediated immune recovery is more likely to involve maintenance of immune cell viability and functional status than an increase in the number of immune cells or mitogen responsiveness.

These findings provide a functional bridge between hematological and downstream immune responses. Although EKP did not restore peripheral leukocyte count or ConA-induced proliferation, it improved the basal viability of splenocytes under immunosuppressive stress. The preserved splenocyte status may have contributed to the recovery of cytokine production, NK cell-associated activity, and humoral immune markers, as described in the following sections.

Taken together, EKP enhanced the basal splenocyte viability under CPA-induced immunosuppressive conditions without promoting mitogen-induced proliferation. This result supports the interpretation that EKP acts primarily through the functional preservation of existing immune cells rather than through their numerical expansion.

### Modulation of Splenic Cytokine Responses Under CPA-induced Immunosuppression

To clarify how EKP influences immune signaling in CPA-induced immunosuppression, splenic cytokine responses were examined, focusing on the key effector cytokines associated with immune activation and inflammatory regulation (Fig. 2B–2E).

CPA treatment markedly disrupted cytokine production in Trial 1. The CPA control group (G2) showed significantly reduced IFN-γ and TNF-α levels compared to the normal control group (G1) (*p* < 0.01), indicating suppression of Th1-associated effector signaling. In contrast, IL-6 levels were strongly elevated in G2, suggesting an excessive inflammatory response to CPA-induced immune stress. Thus, CPA did not simply suppress cytokine production; it produced an imbalanced cytokine profile characterized by weakened effector signaling and enhanced inflammatory activation.

Administration of EKP reversed this pattern in a dose-dependent manner. IFN-γ and TNF-α levels increased progressively across EKP-treated groups, reaching significance from the low dose for IFN-γ (G3; *p* < 0.05) and from the mid dose for TNF-α (G4; *p* < 0.01), with the high-dose group (G5) reaching or exceeding the levels observed in G1. Concurrently, EKP significantly reduced the CPA-induced increase in IL-6 levels at the mid and high doses (both *p* < 0.01). These bidirectional changes indicated that EKP does not act as a nonspecific cytokine enhancer. Instead, EKP restored the suppressed effector cytokines while attenuating excessive IL-6 signaling, suggesting a shift toward a more balanced immune response.

The opposing regulation of effector cytokines and IL-6 is mechanistically consistent rather than contradictory. Under CPA-induced injury, the suppression of IFN-γ and TNF-α reflects impaired effector immune function, whereas the elevation of IL-6 arises from cellular damage, as IL-6 is released in response to tissue injury and functions as a damage-associated inflammatory mediator [21]. Therefore, the restoration of immune homeostasis requires bidirectional correction: recovery of the suppressed effector cytokines together with attenuation of the excessive, damage-driven IL-6 response. The observation that EKP simultaneously restored IFN-γ and TNF-α while lowering IL-6 indicates that it did not act as a nonspecific stimulant but rather re-established a balanced cytokine environment. This rebalanced state, characterized by restored IL-12 and IFN-γ together with normalized IL-6, provides the immunological context required for the recovery of downstream effector functions, including the NK cell-associated activity described in the following section.

Additional support for this interpretation was obtained from Trial 2, in which the IL-12 p70 levels were quantifiable. Although CPA did not significantly reduce IL-12 p70 levels relative to G1, EKP administration significantly increased IL-12 p70 levels at the highest dose (G5; *p* < 0.05; Fig. 2E). Because IL-12 is an upstream regulator of IFN-γ production and NK cell activation [22], this response suggests that EKP may support innate immune priming through the IL-12–IFN-γ-related axis. This finding was consistent with the recovery of IFN-γ observed in Trial 1 and provides a mechanistic link to the NK cell-associated response described in the following section.

These cytokine results are particularly meaningful when interpreted together with the hematological and splenocyte findings. As shown by hematological and splenocyte proliferation data, EKP did not restore peripheral leukocyte numbers or ConA-induced proliferative responses. Nevertheless, EKP restored IFN-γ and TNF-α production, increased IL-12 p70 levels, and reduced excessive IL-6 levels. This indicates that EKP improves the immune signaling capacity within the remaining immune cell population, rather than through numerical immune cell expansion.

From a mechanistic perspective, this cytokine rebalancing may be related to the recognition of paramylon, the β-1,3-glucan component of EKP, by pattern recognition receptors such as Dectin-1 on myeloid cells [23, 24]. Such receptor-mediated recognition could contribute to IL-12 production and subsequent IFN-γ-associated immune activation. In parallel, the suppression of IL-6 suggests that EKP may help limit excessive inflammatory signaling under CPA-induced stress [21]. However, because receptor-specific signaling was not directly examined in this study, this pathway should be interpreted as a plausible mechanism rather than a confirmed conclusion.

The immunomodulatory activities of EKP, purified paramylon, and yeast- or fungus-derived β-glucans are expected to differ according to both structure and composition. β-Glucan bioactivity is governed by backbone linkage, branching, molecular weight, and solubility, and paramylon lies at one extreme of this spectrum as a linear, unbranched, highly crystalline β-1,3-glucan, whereas yeast- and fungus-derived β-glucans carry β-1,6 side chains. Since ligand size, branching, and solubility are established determinants of Dectin-1-mediated responses [23, 24], these differences likely alter the efficiency of receptor engagement rather than invoking distinct signaling pathways. EKP differs further from purified paramylon in retaining non-glucan constituents such as proteins and amino acids, which may contribute additively. Consistent with this, EKP and β-glucan produced broadly comparable effects across most endpoints in the present study, diverging mainly in ConA-induced proliferation, which was significantly reduced by β-glucan but not by EKP. Direct side-by-side comparisons under identical conditions are required to definitively resolve these differences.

Taken together, EKP rebalanced CPA-disrupted cytokine responses by restoring IFN-γ and TNF-α production, increasing IL-12 p70 levels, and suppressing excessive IL-6 production. These coordinated cytokine changes provide a functional basis for the recovery of NK cell-associated activity, as described in the next section.

### EKP Restored NK Cell-Associated Functional Responsiveness

To determine whether the observed cytokine changes were accompanied by functional immune recovery, NK cell activity was assessed based on IFN-γ secretion following activator stimulation (Fig. 2F).

CPA treatment markedly suppressed NK cell-associated activity. The CPA control group (G2) showed an approximately 86% reduction compared to the normal control (G1) (*p* < 0.01), indicating severe impairment of innate immune responsiveness under CPA-induced immunosuppressive conditions [9]. In contrast, EKP administration restored the NK cell-associated activity in a dose-dependent manner. Significant recovery was observed across all EKP-treated groups, with a modest increase in the low-dose group (G3; *p* < 0.05) and stronger recovery in the mid- and high-dose groups (G4 and G5; both *p* < 0.01) as well as in the positive control group (G6; *p* < 0.01). These results indicate a graded dose-related recovery of NK cell-associated functional responsiveness.

This functional recovery is consistent with the cytokine profiles described above. EKP increased IL-12 p70 levels and restored IFN-γ production, both of which are closely associated with NK cell activation and cytotoxic immune responses [22]. Therefore, the recovery of activator-induced IFN-γ secretion supports the interpretation that EKP-mediated cytokine rebalancing was translated into improved innate immune function.

Notably, this improvement occurred despite the absence of peripheral leukocyte recovery, as previously described. EKP did not significantly increase circulating WBC or lymphocyte counts but restored NK cell-associated activity across all doses tested. This dissociation further supports the idea that EKP enhances the functional responsiveness of existing immune cells, rather than increasing their circulating numbers, as demonstrated by the hematological data (Supplementary Table S1).

Taken together, EKP restored NK cell-associated functional activity across all doses tested in CPA-treated mice, with the greatest effects observed at mid and high doses. These findings provide functional evidence that EKP-mediated cytokine rebalance is accompanied by improved innate immune responsiveness under CPA-induced immunosuppressive stress.

### EKP Restored Humoral Immune Markers

To determine whether EKP-mediated immune restoration extended beyond cytokine- and NK cell-associated responses, serum immunoglobulin levels were quantified in Trial 2 (Fig. 3A–3C). CPA treatment markedly suppressed the expression of humoral immune markers. The CPA control group (G2) showed significantly lower IgG, IgA, and IgM levels compared to the normal control group (G1) (all *p* < 0.01), indicating broad impairment of B cell-associated antibody responses under CPA-induced immunosuppressive stress [2].

EKP restored the levels of all three immunoglobulin classes in a dose-dependent manner. IgG showed the most consistent recovery, with all EKP-treated groups showing a significant increase compared to G2 (G3: *p* < 0.05; G4, G5, G6: *p* < 0.01) and reaching levels statistically comparable to G1 (Fig. 3A). IgM levels were significantly increased in the mid-dose EKP group (G4; *p* < 0.05) and the positive control group (G6; *p* < 0.05), indicating partial recovery of primary antibody-associated responses (Fig. 3C). IgA exhibited a clear dose-dependent increase, with significant recovery in groups G5 (*p* < 0.01) and G6 (*p* < 0.01), whereas the low- and mid-dose groups showed non-significant increasing trends (Fig. 3B). These results indicate that EKP supported recovery across multiple immunoglobulin classes, although the dose at which significance was reached differed among them.

Recovery of IgA is particularly relevant because EKP is administered orally. IgA production is closely associated with gut-associated lymphoid tissue (GALT), and β-1,3-glucan particles can be taken up by intestinal dendritic cells in Peyer’s patches [25], which are important sites for IgA class switching and mucosal immune induction [26]. Thus, increased serum IgA levels may reflect a possible link between oral EKP exposure, mucosal immune activation, and systemic humoral recovery. However, because intestinal immune tissues were not directly examined in this study, this gut-associated immune pathway should be considered a plausible interpretation rather than a confirmed mechanism.

When viewed together with the cytokine- and NK cell-associated responses described above, the immunoglobulin data show that EKP-mediated immune recovery is not limited to innate immune signaling. EKP also improved antibody-related immune markers, with IgG responding to the lowest dose, and IgA and IgM showing class-specific dose thresholds. Importantly, this humoral recovery occurred despite the absence of measurable hematopoietic expansion, further supporting the interpretation that EKP enhances immune function without measurably increasing the number of circulating immune cells.

Taken together, EKP restored serum IgG, IgM, and IgA levels in CPA-treated mice, with clear effects at mid and high doses. These findings indicate that EKP supports humoral immune recovery under established immunosuppressive conditions and suggest that oral EKP may also engage mucosal immune pathways. However, further analysis of GALT-associated tissues is required to confirm this possibility.

### Structural Validation and Integrated Mechanism of Functional Immune Restoration Without Measurable Leukocyte Expansion

To determine whether the observed functional immune recovery was accompanied by tissue restoration, splenic histopathology was examined in Trial 2 (Fig. 4). The CPA control group (G2) exhibited severe splenic architectural disruption characterized by marked white pulp atrophy, loss of germinal centers, and extensive lymphocyte depletion. These histological changes were consistent with the hematological suppression and decreased immunoglobulin levels observed in the same trial as well as with the impaired cytokine responses and reduced NK cell-associated activity described in Trial 1, confirming that CPA induces substantial splenic immune injury.

In contrast, EKP administration resulted in dose-dependent restoration of the splenic architecture. The low-dose group (G3) showed partial recovery of white pulp organization, whereas the mid-dose group (G4) showed clearer structural boundaries and early germinal center formation. Notably, the high-dose group (G5) showed well-organized white pulp regions and developed germinal centers, displaying splenic morphology similar to that of the control group (G1). The positive control group (G6) showed a similar pattern of structural recovery. These findings indicate that EKP-mediated immune recovery is accompanied by the restoration of lymphoid tissue organization, particularly at higher doses.

The reappearance of germinal center-like structures is particularly relevant to humoral immune recovery, as described above. Germinal centers are key sites for B cell activation, affinity maturation, and antibody production [27]. Therefore, histological recovery of the germinal center structure was consistent with the restoration of serum IgG, IgM, and IgA levels after EKP treatment. This agreement between splenic architecture and immunoglobulin recovery suggests that EKP supports not only functional immune readouts but also the tissue-level organization required for coordinated immune responses.

When integrated with preceding findings, the present findings support a non-proliferative immune restoration framework. EKP did not significantly restore peripheral leukocyte numbers or ConA-induced splenocyte proliferation. Nevertheless, it enhanced basal splenocyte viability, rebalanced cytokine responses, restored NK cell-associated functional responsiveness, improved serum immunoglobulin levels, and promoted splenic architectural recovery. Thus, EKP-associated immune restoration cannot be explained simply by hematopoietic stimulation or numerical expansion of immune cells. Instead, EKP appears to improve immune competence by preserving the functional status of the remaining immune cells and supporting the reorganization of immune tissues under CPA-induced immunosuppressive stress.

From a mechanistic perspective, this integrated response may be related to the β-1,3-glucan component of EKP, paramylon. Recognition of β-1,3-glucan by pattern recognition receptors such as Dectin-1 on myeloid cells [23] could promote IL-12 production, thereby supporting an IL-12 –IFN-γ-related pathway [22], consistent with the observed recovery of IFN-γ production and NK cell-associated activity. Simultaneously, the suppression of excessive IL-6 signaling may have contributed to a more balanced inflammatory environment, whereas splenic architectural recovery and germinal center reformation may have contributed to the restoration of antibody production. These coordinated responses provide a plausible link between innate immune signaling, humoral immune recovery, and splenic tissue restoration.

However, this proposed mechanism should be interpreted with caution. This study did not directly examine receptor-specific signaling, immune cell subset dynamics, or gut-associated lymphoid tissues. In addition, because whole EKP rather than purified paramylon was used, the specific contribution of paramylon relative to other *Euglena*-derived constituents could not be delineated, and a direct comparison of purified paramylon and whole EKP represents an important direction for future mechanistic studies. Therefore, although these data support a functional and structural immune restoration framework, additional mechanistic studies are required to confirm the specific cellular and molecular pathways involved.

Overall, EKP promoted the functional and structural recovery of the immune status in CPA-treated mice without measurable peripheral leukocyte expansion. The convergence of splenocyte viability, cytokine rebalancing, NK cell-associated activity, immunoglobulin restoration, and splenic architectural recovery supports a non-proliferative immune restoration framework in which EKP improves immune competence primarily through functional preservation and immune tissue reorganization, rather than through expansion of circulating leukocyte populations.

## Conclusion

This study evaluated the immunorestorative effects of EKP in a dual-temporal CPA-induced immunosuppression model. Despite the persistent depletion of circulating leukocytes across both the acute and established immunosuppressive phases, EKP administration was associated with functional and structural evidence of immune recovery, particularly at mid and high doses.

Specifically, EKP increased basal splenocyte viability under CPA-induced stress and modulated the splenic cytokine profile by restoring IFN-γ and TNF-α production while reducing excessive IL-6 levels. EKP also improved NK cell-associated activity across all doses in Trial 1 and significantly increased IL-12 p70 levels at the highest dose in Trial 2; although these indicators were assessed in separate trials, their concordant directions are consistent with the engagement of an IL-12–IFN-γ–related innate immune response. These innate, cellular, and humoral immune changes were accompanied by the recovery of serum IgG, IgM, and IgA levels, with an increase in IgA levels, suggesting a possible contribution of GALT activation following oral EKP administration. Splenic histopathology further supported these functional findings by showing dose-related improvements in white pulp organization and germinal center formation.

Collectively, these findings indicate that EKP may improve immune function through a non-proliferative immunorestorative mechanism, restoring the functional competence of existing immune cell populations, rather than increasing the number of immune cells. These results provide preclinical evidence supporting the immune-enhancing potential of EKP and may serve as foundational data for future human intervention studies aimed at validating its functionality as a food ingredient. Future studies are required to further elucidate the molecular mechanisms underlying this immunomodulatory effect.

## Supplemental Materials

Supplementary data for this paper are available on-line only at http://jmb.or.kr.



## Figures and Tables

**Fig. 1:**
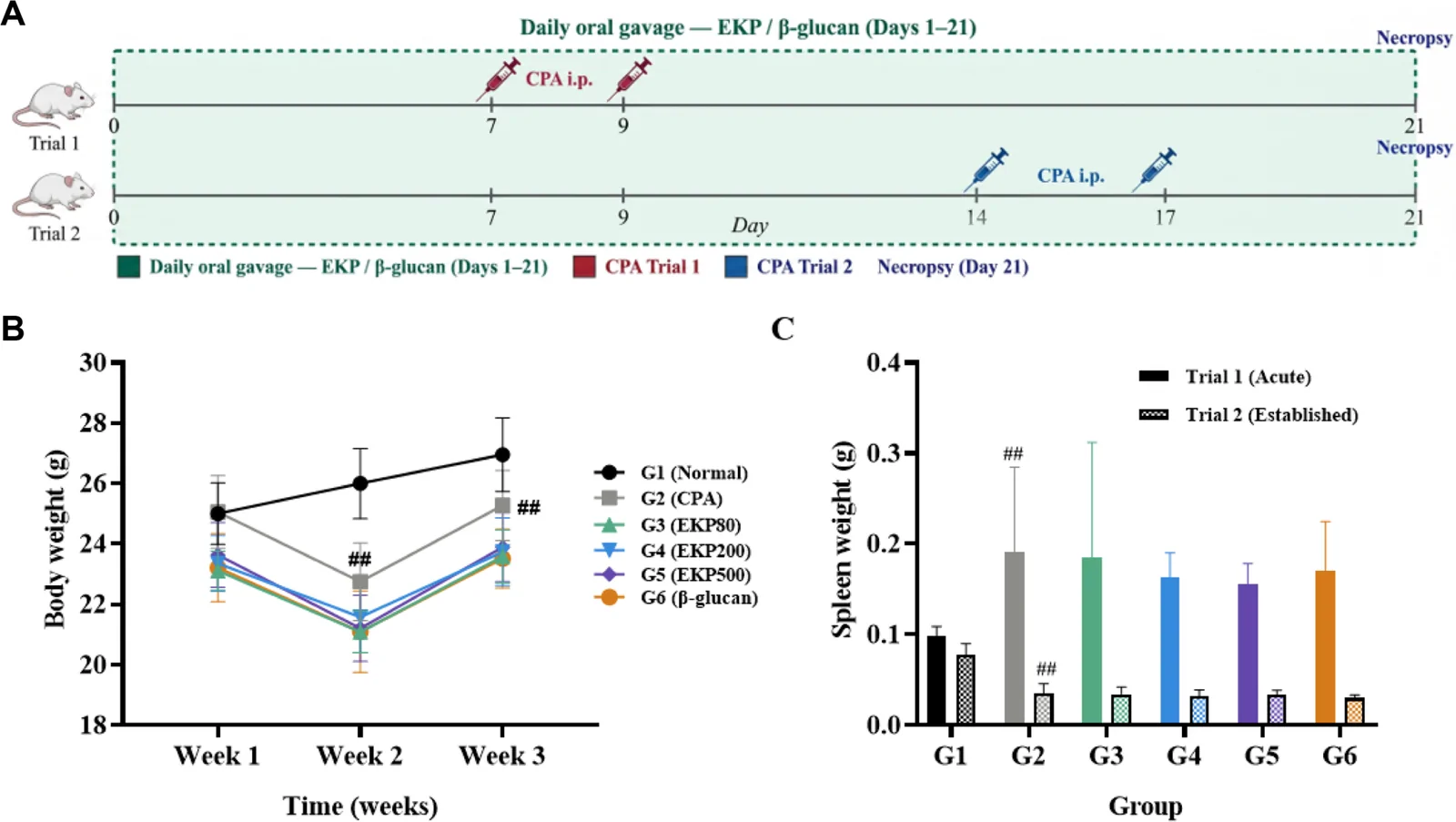
Experimental design, body weight changes, and spleen weight in CPA-induced immunosuppressed mice. (**A**) Schematic overview of dual-temporal experimental design. *Euglena gracilis* EK powder (EKP) and β-glucan were administered via oral gavage for 21 consecutive days. Cyclophosphamide (CPA; 150 mg/kg) was administered intraperitoneally on days 7 and 9 in Trial 1 (acute phase) and on days 14 and 17 in Trial 2 (established phase). Animals were necropsied on day 21. (**B**) Body weight changes during 21-day treatment period — Trial 1. Data are expressed as mean ± SD (n = 10 per group) and were analyzed by two-way repeated measures ANOVA with Geisser-Greenhouse correction followed by Dunnett's multiple comparisons test against G1. ^##^*p* < 0.01 vs. G1 (normal control), shown for G2. All EKP-treated groups (G3–G5) and positive control group (G6) differed significantly from G1 at all three time points (*p* < 0.05–0.01), including at week 1 prior to CPA administration, reflecting baseline weight imbalance at group allocation. (C) Absolute spleen weights at necropsy (day 21) in Trial 1 (filled bars) and Trial 2 (hatched bars). Data are expressed as mean ± SD (n = 10 per group). ^##^*p* < 0.01 vs. G1 (normal control). G1, normal control; G2, CPA control; G3, EKP 80 mg/kg; G4, EKP 200 mg/kg; G5, EKP 500 mg/kg; G6, β-glucan 400 mg/kg.

**Fig. 2:**
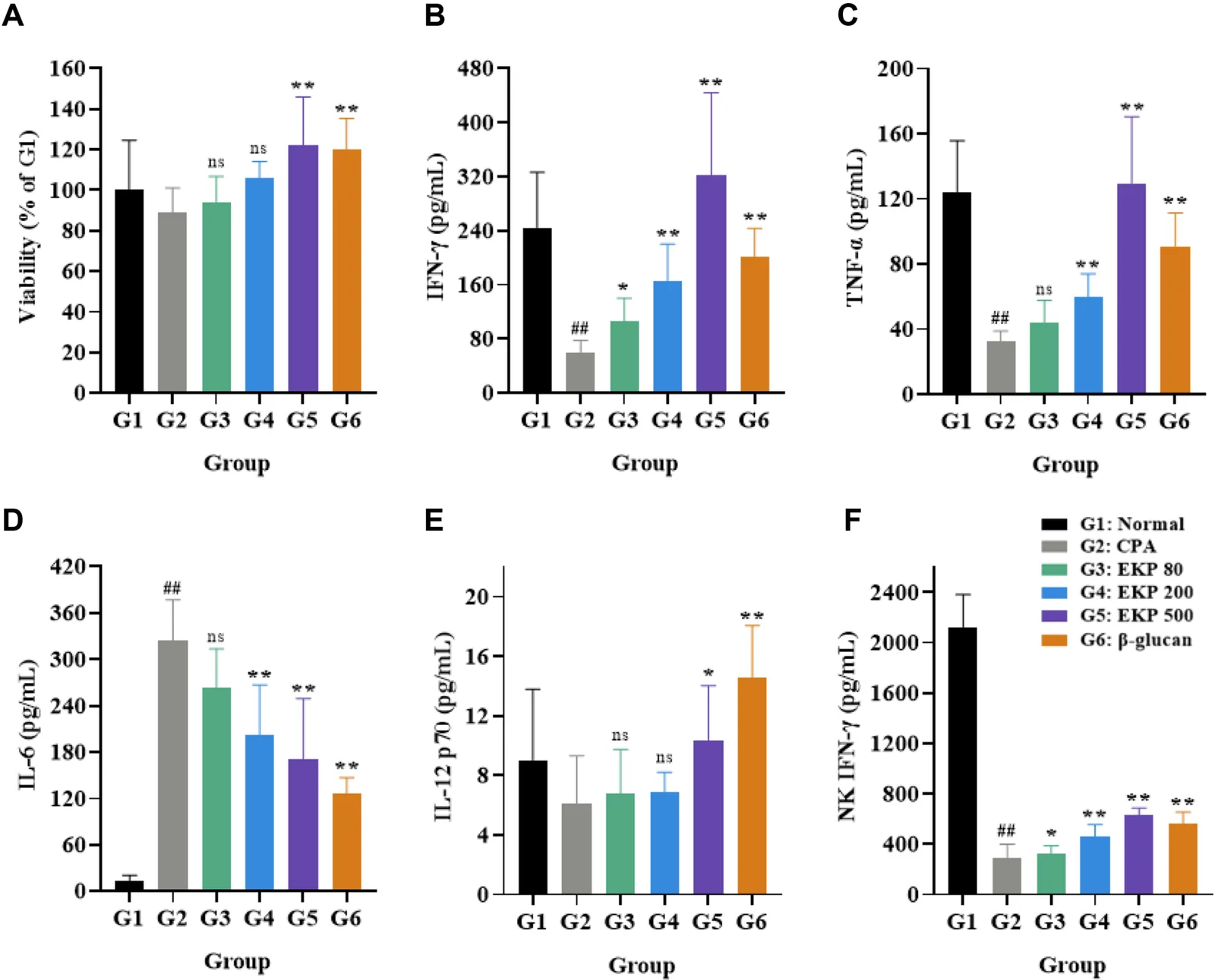
Splenocyte-based immune functional responses following EKP administration. (**A**) Basal splenocyte viability assessed under unstimulated conditions using CCK-8 assay (Trial 1), expressed as a percentage relative to the normal control group (G1). (**B**) IFN-γ, (**C**) TNF-α, and (**D**) IL-6 concentrations in culture supernatants quantified by ELISA following anti-CD3/CD28 monoclonal antibody stimulation (1 μg/well each; 48 h; Trial 1). (**E**) IL-12 p70 concentration following anti-CD3/CD28 stimulation (48 h; Trial 2); IL-12 p70 was below the limit of quantification in Trial 1 so was only quantified in Trial 2, and CPA treatment did not significantly reduce IL-12 p70 levels relative to G1. (**F**) NK cell-associated activity assessed by IFN-γ secretion following NK cell activator stimulation (24 h; Trial 1). Data are expressed as mean ± SD (n = 8 per group). ^##^*p* < 0.01 vs. G1 (normal control); *p < 0.05, **p < 0.01 vs. G2 (CPA control). ns, not significant. Comparisons between G2 and G6 were performed by independent samples t-test; comparisons between G2 and G3–G5 were performed by multiple comparison procedures. G1, normal control; G2, CPA control; G3, EKP 80 mg/kg; G4, EKP 200 mg/kg; G5, EKP 500 mg/kg; G6, β-glucan 400 mg/kg.

**Fig. 3:**
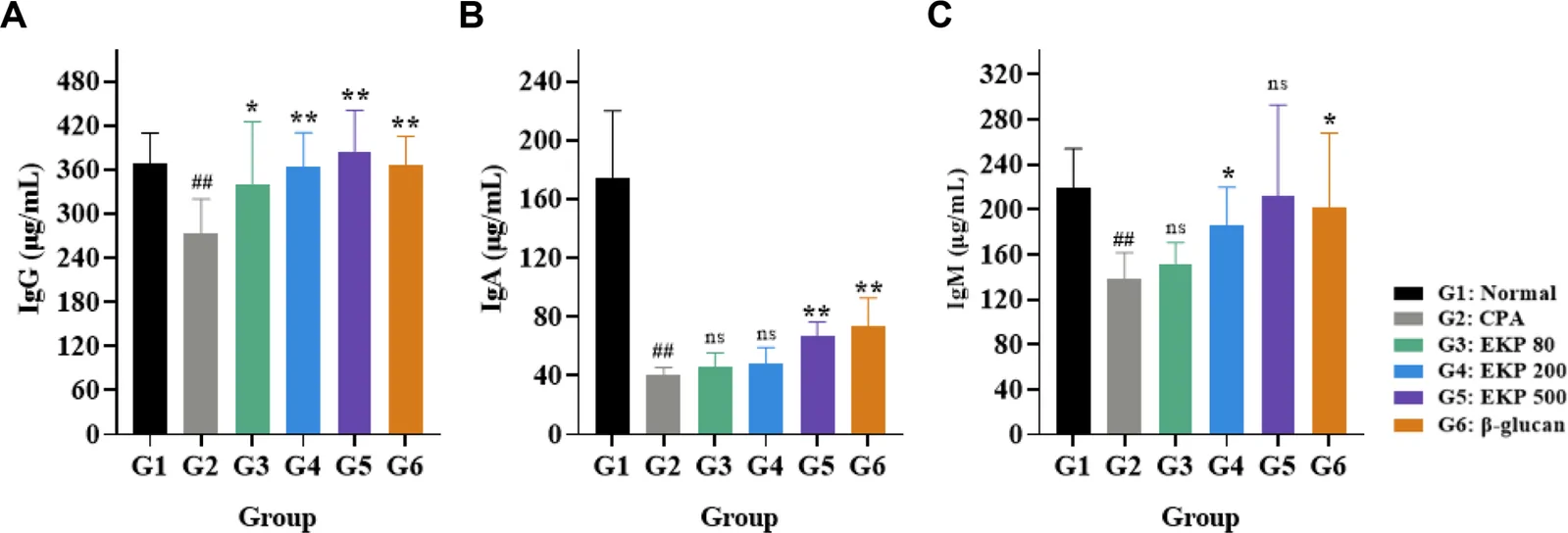
Serum immunoglobulin levels in Trial 2. (**A**) IgG, (**B**) IgA, and (**C**) IgM concentrations in serum collected on day 21 (Trial 2) were quantified by ELISA. Data are expressed as mean ± SD (n = 10 per group). ^##^*p* < 0.01 vs. G1 (normal control); *p < 0.05, **p < 0.01 vs. G2 (CPA control). ns, not significant. Comparisons between G2 and G6 were performed by independent samples t-test; comparisons between G2 and G3–G5 were performed by multiple comparison procedures. G1, normal control; G2, CPA control; G3, EKP 80 mg/kg; G4, EKP 200 mg/kg; G5, EKP 500 mg/kg; G6, β-glucan 400 mg/kg.

**Fig. 4:**
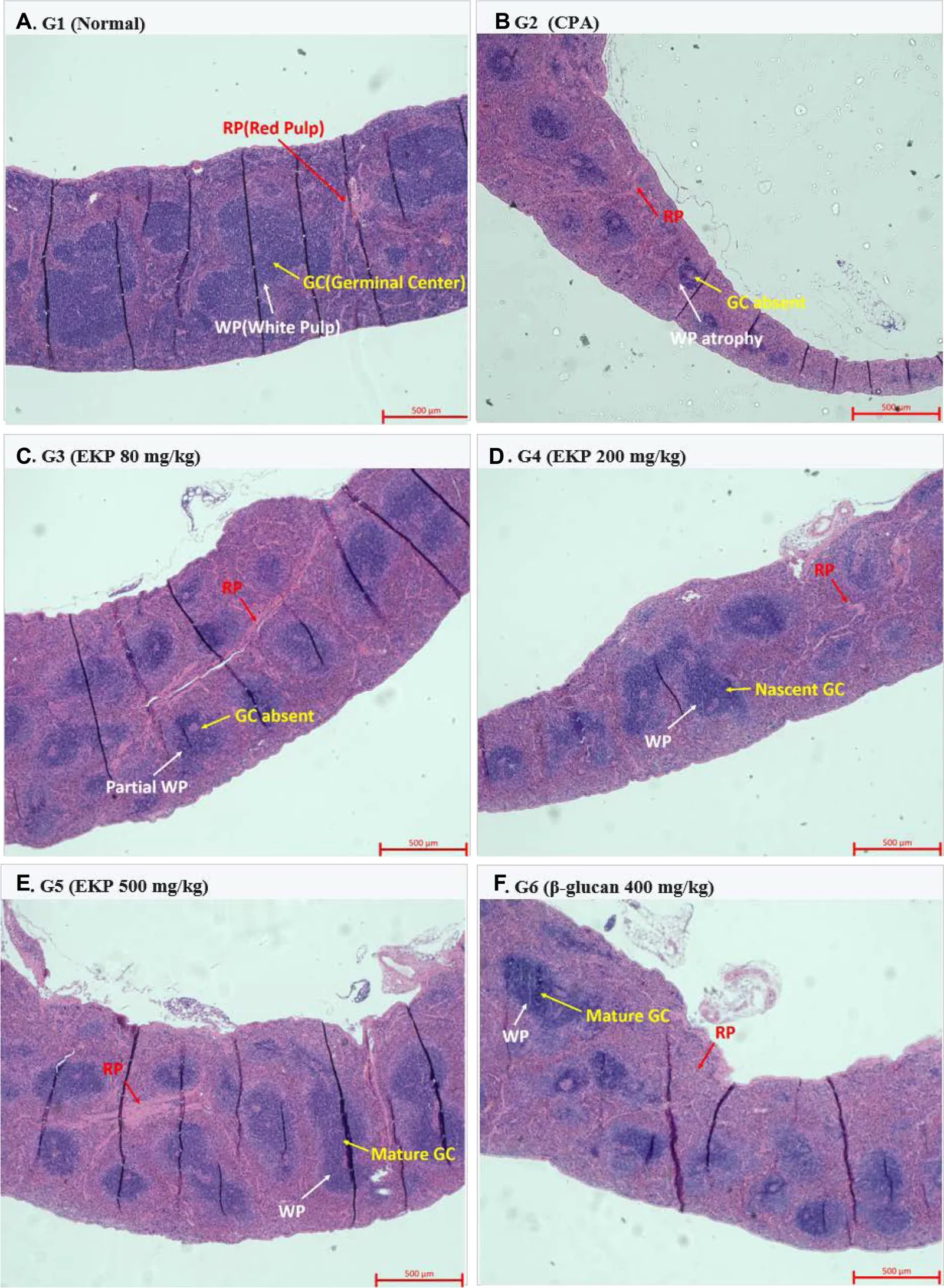
Representative splenic histopathology in CPA-induced immunosuppressed mice. Representative splenic sections (*n* = 2 per group; 4 μm; H&E staining; blinded evaluation by a board-certified veterinary pathologist). (**A**) G1 (normal control) showed well-defined white pulp (WP), red pulp (RP), and mature germinal center (GC). (**B**) G2 (CPA control) exhibited marked WP atrophy, near-complete GC loss, and extensive lymphocyte depletion. EKP administration produced dose-dependent structural recovery: (**C**) G3 showed partial WP restoration with no clear GC; (**D**) G4 exhibited nascent GC formation with clearer WP boundaries; (**E**) G5 displayed splenic architecture closely resembling G1, with well-organized WP and mature GC. (**F**) G6 (β-glucan 400 mg/kg) showed comparable structural restoration to G5. Scale bar = 500 μm. White arrows, WP; yellow arrows, GC; red arrows, RP.

## References

[1] Emadi A, Jones RJ, Brodsky RA (2009). Cyclophosphamide and cancer: golden anniversary. Nat. Rev. Clin. Oncol..

[2] Ahlmann M, Hempel G (2016). The effect of cyclophosphamide on the immune system: implications for clinical cancer therapy. Cancer Chemother. Pharmacol..

[3] Gissibl A, Sun A, Care A, Nevalainen H, Sunna A (2019). Bioproducts From *Euglena gracilis*: Synthesis and Applications. Front. Bioeng. Biotechnol..

[4] Jung WS, Jung Y, Kim JY, Bae EH, Choi Y-E (2026). Co-utilization of bread waste and harmful algal bloom-derived cyanobacterial biomass for *Euglena gracilis* cultivation: Turning waste and pollution into resources. Algal Res..

[5] Jeon MS, Oh J-J, Kim JY, Han S-I, Sim SJ, Choi Y-E (2019). Enhancement of growth and paramylon production of *Euglena gracilis* by co-cultivation with Pseudoalteromonas sp. MEBiC 03485. Bioresour. Technol..

[6] Goodridge HS, Wolf AJ, Underhill DM (2009). Beta-glucan recognition by the innate immune system. Immunol. Rev..

[7] Volman JJ, Ramakers JD, Plat J (2008). Dietary modulation of immune function by beta-glucans. Physiol. Behav..

[8] Brown GD, Herre J, Williams DL, Willment JA, Marshall AS, Gordon S (2003). Dectin-1 mediates the biological effects of beta-glucans. J. Exp. Med..

[9] Yang H, Choi K, Kim KJ, Park SY, Jeon JY, Kim BG (2022). Immunoenhancing Effects of *Euglena gracilis* on a Cyclophosphamide-Induced Immunosuppressive Mouse Model. J. Microbiol. Biotechnol..

[10] Jo SH, Jo KA, Park SY, Kim JY (2024). Unveiling Immunomodulatory Effects of *Euglena gracilis* in Immunosuppressed Mice: Transcriptome and Pathway Analysis. J. Microbiol. Biotechnol..

[11] National Research Council. 2011. *Guide for the Care and Use of Laboratory Animals: Eighth Edition*, The National Academies Press, Washington, DC.

[12] Jo KA, Kim KJ, Park SY, Jeon JY, Hwang JE, Kim JY (2023). Evaluation of the Effects of *Euglena gracilis* on Enhancing Immune Responses in RAW264.7 Cells and a Cyclophosphamide-Induced Mouse Model. J. Microbiol. Biotechnol..

[13] Turner PV, Brabb T, Pekow C, Vasbinder MA (2011). Administration of substances to laboratory animals: routes of administration and factors to consider. J. Am. Assoc. Lab. Anim. Sci..

[14] Kruisbeek AM. 2001. Isolation of mouse mononuclear cells. *Curr. Protoc. Immunol.* Chapter **3:** Unit 3 1. https://doi.org/10.1002/0471142735.im0301s39. 10.1002/0471142735.im0301s39 18432779

[15] Tominaga H, Ishiyama M, Ohseto F, Sasamoto K, Hamamoto T, Suzuki K (1999). A water-soluble tetrazolium salt useful for colorimetric cell viability assay. Anal. Commun..

[16] Park SY, Kim KJ, Jo SM, Jeon JY, Kim BR, Hwang JE (2023). *Euglena gracilis* (*Euglena*) powder supplementation enhanced immune function through natural killer cell activity in apparently healthy participants: A randomized, double-blind, placebo-controlled trial. Nutr. Res..

[17] Elmore SA (2006). Enhanced histopathology of the spleen. Toxicol. Pathol..

[18] Wang Y, Meng Q, Qiao H, Jiang H, Sun X (2009). Role of the spleen in cyclophosphamide-induced hematosuppression and extramedullary hematopoiesis in mice. Arch. Med. Res..

[19] Huyan XH, Lin YP, Gao T, Chen RY, Fan YM (2011). Immunosuppressive effect of cyclophosphamide on white blood cells and lymphocyte subpopulations from peripheral blood of Balb/c mice. Int. Immunopharmacol..

[20] Salem ML, Al-Khami AA, El-Nagaar SA, Zidan AA, Al-Sharkawi IM, Marcela Diaz-Montero C (2012). Kinetics of rebounding of lymphoid and myeloid cells in mouse peripheral blood, spleen and bone marrow after treatment with cyclophosphamide. Cell Immunol..

[21] Tanaka T, Narazaki M, Kishimoto T (2014). IL-6 in inflammation, immunity, and disease. Cold Spring Harb. Perspect. Biol..

[22] Trinchieri G (2003). Interleukin-12 and the regulation of innate resistance and adaptive immunity. Nat. Rev. Immunol..

[23] Taylor PR, Tsoni SV, Willment JA, Dennehy KM, Rosas M, Findon H (2007). Dectin-1 is required for beta-glucan recognition and control of fungal infection. Nat. Immunol..

[24] Reid DM, Gow NA, Brown GD (2009). Pattern recognition: recent insights from Dectin-1. Curr. Opin. Immunol..

[25] De Jesus M, Ostroff GR, Levitz SM, Bartling TR, Mantis NJ (2014). A population of Langerin-positive dendritic cells in murine Peyer's patches involved in sampling β-glucan microparticles. PLoS One.

[26] Mowat AM, Agace WW (2014). Regional specialization within the intestinal immune system. Nat. Rev. Immunol..

[27] Mesin L, Ersching J, Victora GD (2016). Germinal Center B Cell Dynamics. Immunity.

