# Supplementary material for: Non-Proliferative Immune Restoration by Euglena gracilis EK Powder in Cyclophosphamide-Induced Immunosuppressed Mice
Source: J Microbiol Biotechnol. 2026 Sep 9;36:e2607031. doi: 10.4014/jmb.2607.07031 (PMC13655588; doi:10.4014/jmb.2607.07031)
Supplement: Supplementary file 1 [file jmb-36-e2607031-supple.pdf]

J. Microbiol. Biotechnol. 2026. 36: e2607031
https://doi.org/10.4014/jmb.2607.07031

Supplementary Figures and Table

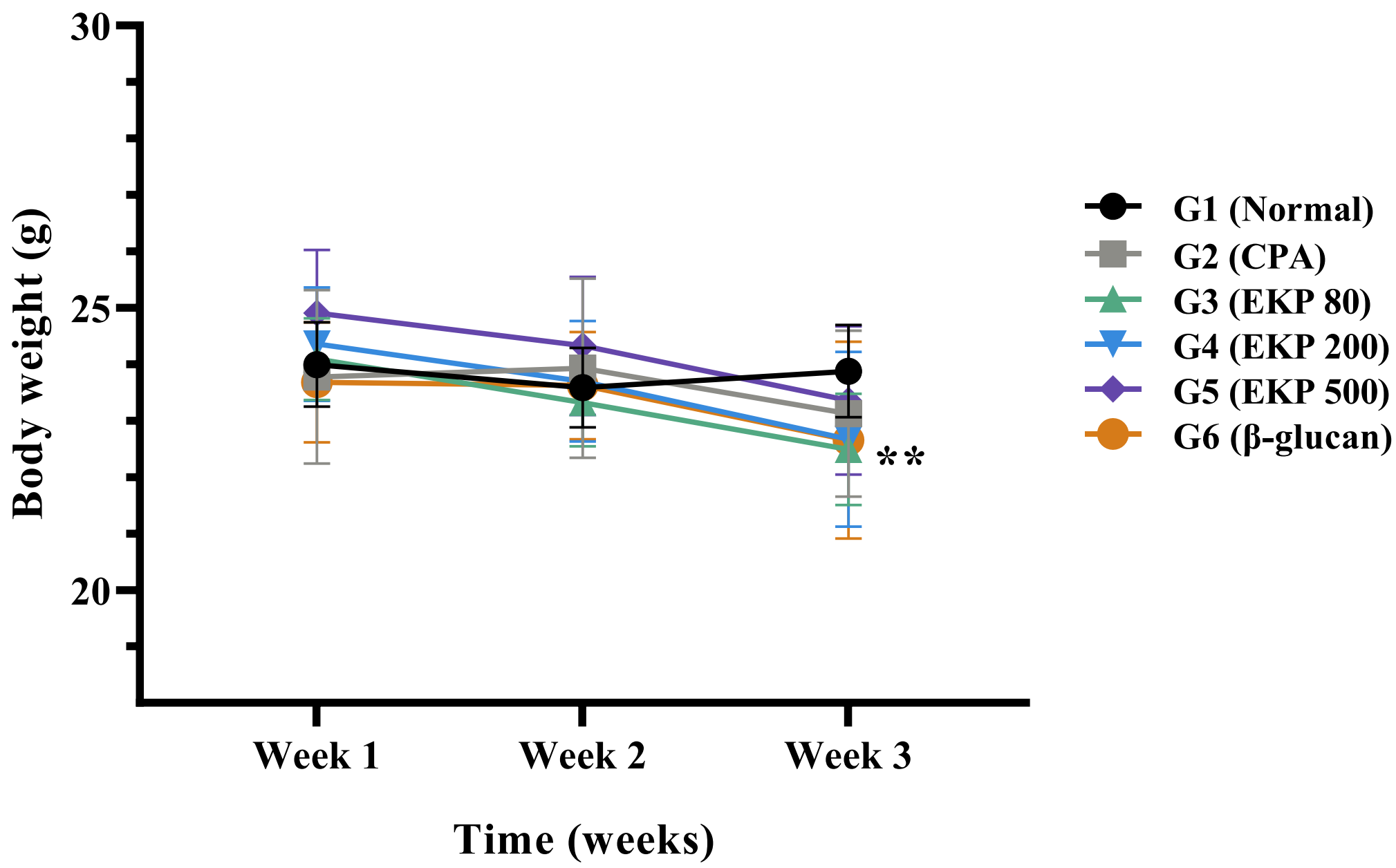

Fig. S1. Body weight changes during the 21-day treatment period — Trial 2.

CPA (150 mg/kg) was administered intraperitoneally on Days 14 and 17. Data are expressed as mean ± SD (n = 10/group). Statistical analysis was performed by two-way repeated measures ANOVA with Geisser-Greenhouse correction followed by Dunnett's multiple comparisons test against G1. No significant differences were detected between G1 and the CPA control group (G2) at any time point, and the only significant inter-group difference was observed between G1 and G3 at Week 3 (**$p = 0.002$). **$p < 0.01$ vs. G1 (normal control). G1, normal control; G2, CPA control; G3, EKP 80 mg/kg; G4, EKP 200 mg/kg; G5, EKP 500 mg/kg; G6, β-glucan 400 mg/kg.

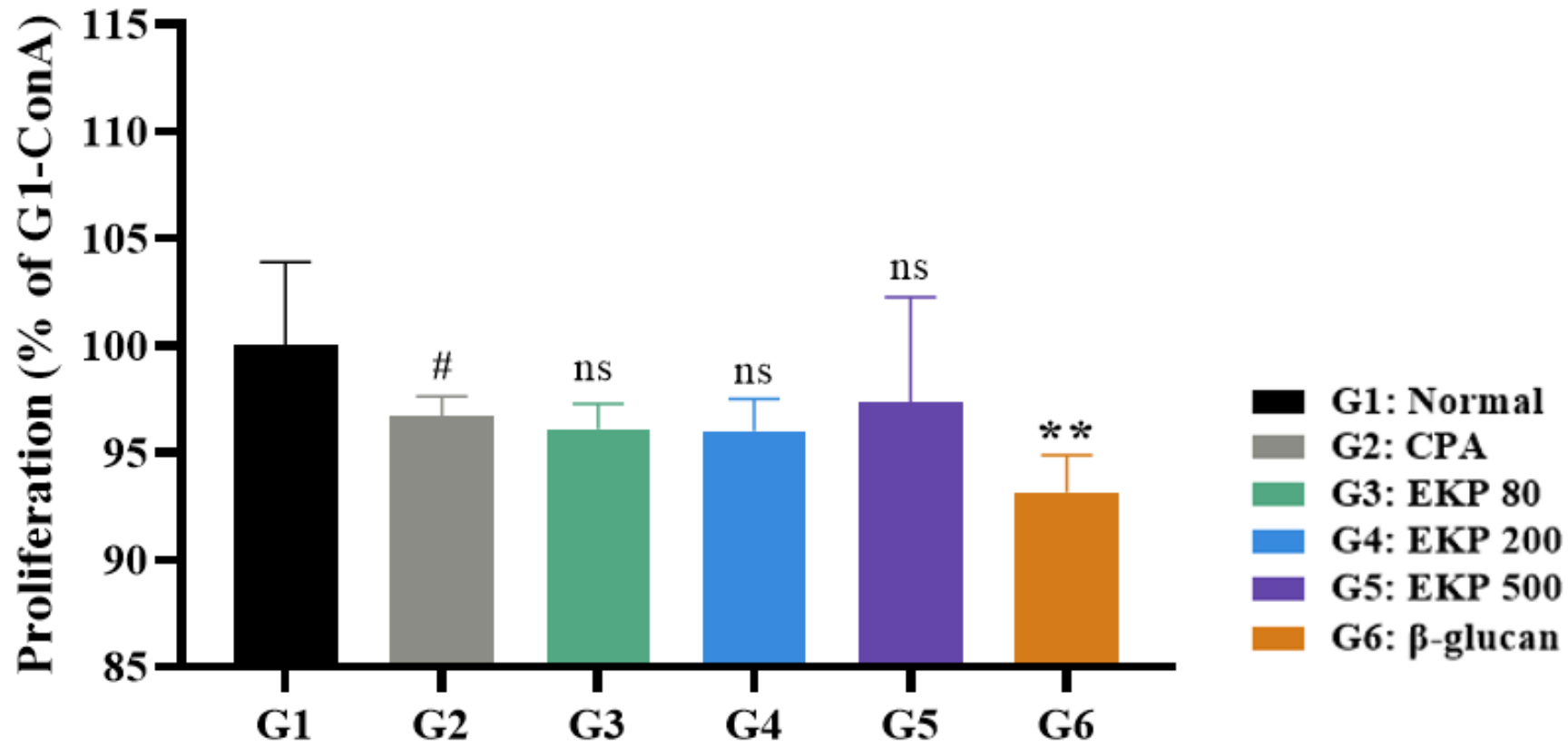

Fig. S2. ConA-stimulated splenocyte proliferation in Trial 1.

Splenocytes (2×10^6/well) were stimulated with ConA (1 μg/mL) for 48 h and viability was assessed by CCK-8 assay. Data are expressed as mean ± SD (n = 8/group) as a percentage relative to the G1-ConA response. $^{\#}p < 0.05$ vs. G1 (normal control); $^{**}p < 0.01$ vs. G2 (CPA control). ns, not significant. G1, normal control; G2, CPA control; G3, EKP 80 mg/kg; G4, EKP 200 mg/kg; G5, EKP 500 mg/kg; G6, β-glucan 400 mg/kg.

Table S1. Peripheral hematological profiles in the dual-temporal CPA-induced immunosuppression model.

Group	WBC (K/μL)	NE (K/μL)	EO (K/μL)	BA (K/μL)	LY (K/μL)	MO (K/μL)	Reti (%)
**Trial 1 (acute phase; CPA on Days 7 and 9)**							
**G1 (Normal)**	5.57 ± 1.70	–	–	–	4.47 ± 1.05	–	–
**G2 (CPA)**	4.57 ± 0.91	–	–	–	1.74 ± 0.62$^{\#\#}$	–	–
**G3 (EKP 80)**	3.28 ± 0.98*	–	–	–	1.05 ± 0.32**	–	–
**G4 (EKP 200)**	2.95 ± 1.37**	–	–	–	0.94 ± 0.42**	–	–
**G5 (EKP 500)**	2.82 ± 0.46**	–	–	–	0.98 ± 0.20**	–	–
**G6 (β-glucan)**	2.32 ± 0.73**	–	–	–	0.73 ± 0.24**	–	–
**Trial 2 (established phase; CPA on Days 14 and 17)**							
**G1 (Normal)**	2.96 ± 0.82	0.587 ± 0.253	0.059 ± 0.020	0.001 ± 0.003	2.34 ± 0.67	0.046 ± 0.019	4.43 ± 1.06
**G2 (CPA)**	0.42 ± 0.37$^{\#\#}$	0.017 ± 0.018$^{\#\#}$	0.000 ± 0.000$^{\#\#}$	0.001 ± 0.003	0.29 ± 0.19$^{\#\#}$	0.110 ± 0.178	0.63 ± 0.15$^{\#\#}$
**G3 (EKP 80)**	0.58 ± 0.26	0.036 ± 0.086	0.000	0.000	0.44 ± 0.12*	0.097 ± 0.118	0.59 ± 0.04
**G4 (EKP 200)**	0.35 ± 0.16	0.009 ± 0.009	0.000	0.001 ± 0.003	0.28 ± 0.09	0.063 ± 0.085	0.57 ± 0.06
**G5 (EKP 500)**	0.34 ± 0.08	0.012 ± 0.008	0.000	0.000	0.27 ± 0.06	0.057 ± 0.059	0.56 ± 0.08
**G6 (β-glucan)**	0.25 ± 0.06	0.014 ± 0.012	0.000	0.000	0.21 ± 0.06	0.029 ± 0.024	0.54 ± 0.05

Peripheral blood was collected on Day 21 and analyzed using an automated hematology analyzer (ADVIA 2120; Siemens Healthineers). In Trial 1, only WBC and lymphocyte counts were determined; a complete differential was obtained in Trial 2. Data are expressed as mean ± SD (Trial 1, *n* = 9–10/group; Trial 2, *n* = 10/group). ^##^*p* < 0.01 vs. G1 (normal control); **p* < 0.05, ***p* < 0.01 vs. G2 (CPA control). Comparisons between G2 and G6 were performed by independent samples *t*-test; comparisons between G2 and G3–G5 were performed by multiple comparison procedures. –, not measured. WBC, white blood cells; NE, neutrophils; EO, eosinophils; BA, basophils; LY, lymphocytes; MO, monocytes; Reti, reticulocytes. G1, normal control; G2, CPA control; G3, EKP 80 mg/kg; G4, EKP 200 mg/kg; G5, EKP 500 mg/kg; G6, β-glucan 400 mg/kg.
